# BCI-Based Consumers' Choice Prediction From EEG Signals: An Intelligent Neuromarketing Framework

**DOI:** 10.3389/fnhum.2022.861270

**Published:** 2022-05-26

**Authors:** Fazla Rabbi Mashrur, Khandoker Mahmudur Rahman, Mohammad Tohidul Islam Miya, Ravi Vaidyanathan, Syed Ferhat Anwar, Farhana Sarker, Khondaker A. Mamun

**Affiliations:** ^1^Advanced Intelligent Multidisciplinary Systems (AIMS) Lab, Institute for Advanced Research (IAR), United International University, Dhaka, Bangladesh; ^2^School of Business and Economics, United International University, Dhaka, Bangladesh; ^3^Department of Mechanical Engineering and UK Dementia Research Institute Care, Research and Technology Centre (DRI-CR&T), Imperial College London, London, United Kingdom; ^4^Institute of Business Administration, University of Dhaka, Dhaka, Bangladesh; ^5^Department of Computer Science and Engineering, University of Liberal Arts Bangladesh, Dhaka, Bangladesh; ^6^Department of Computer Science & Engineering, United International University, Dhaka, Bangladesh

**Keywords:** Brain Computer Interface, neuromarketing, machine learning, electroencephalography, consumer behavior, pattern recognition, consumer neuroscience

## Abstract

Neuromarketing relies on Brain Computer Interface (BCI) technology to gain insight into how customers react to marketing stimuli. Marketers spend about *$*750 billion annually on traditional marketing camping. They use traditional marketing research procedures such as Personal Depth Interviews, Surveys, Focused Group Discussions, and so on, which are frequently criticized for failing to extract true consumer preferences. On the other hand, Neuromarketing promises to overcome such constraints. This work proposes a machine learning framework for predicting consumers' purchase intention (PI) and affective attitude (AA) from analyzing EEG signals. In this work, EEG signals are collected from 20 healthy participants while administering three advertising stimuli settings: product, endorsement, and promotion. After preprocessing, features are extracted in three domains (time, frequency, and time-frequency). Then, after selecting features using wrapper-based methods Recursive Feature Elimination, Support Vector Machine is used for categorizing positive and negative (AA and PI). The experimental results show that proposed framework achieves an accuracy of 84 and 87.00% for PI and AA ensuring the simulation of real-life results. In addition, AA and PI signals show N200 and N400 components when people tend to take decision after visualizing static advertisement. Moreover, negative AA signals shows more dispersion than positive AA signals. Furthermore, this work paves the way for implementing such a neuromarketing framework using consumer-grade EEG devices in a real-life setting. Therefore, it is evident that BCI-based neuromarketing technology can help brands and businesses effectively predict future consumer preferences. Hence, EEG-based neuromarketing technologies can assist brands and enterprizes in accurately forecasting future consumer preferences.

## 1. Introduction

Neuromarketing is a subfield of marketing research that investigates customers' cognitive and emotive responses to promoted products or services. It is an emerging multidisciplinary area that brings together neuroscience, technology, and traditional marketing. Neuromarketing uses Brain-Computer Interface (BCI) technologies to gain insight into consumers' preferences and purchase intention triggered by marketing stimuli. Furthermore, one of the main objectives of marketing professional is to present their advertisement in such a way that the intended consumer response is elicited. They spend $750 billion or more every year on marketing, promotion, and advertising to achieve this (Guttmann, 2021). Hence, there is a significant motivation to investigate opportunities for targeting the appropriate market segments and customers.

Traditional research methods rely on consumers filling out questionnaires, focus group discussion, or one-on-one interviews to determine their attitudes toward products, mostly on post-facto basis (Hulland et al., 2018). Although these approaches are simple, they oftentimes fail to reflect the true state of mind of the customers, primarily because of the limitations associated with self-reported questionnaire surveys (Rawnaque et al., 2020). Neuromarketing, on the other hand, solves these issues by focusing on capturing the in-person response by taking into account brain response. As a result, there is a need for technology-enabled autonomous prediction of consumer preferences.

In the last 20 years, researchers proposed several automatic approaches with some of these considering the neurological mechanisms that drive marketing decision-making and contribute to the rapidly expanding field of neuromarketing research. In neuromarketing studies, researchers use biometric responses such as facial expression (Filipović et al., 2020), eye tracking (Khushaba et al., 2013), functional magnetic resonance imaging (fMRI) (Hsu and Cheng, 2018), galvanic skin response (Ohira and Hirao, 2015), and electroencephalography (EEG) (Golnar-Nik et al., 2019), magnetoencephalograpy (MEG) (Hege et al., 2014) to extract customers' insights. Previously, the neuromarketing community was primarily interested in fMRI, which assesses cerebral blood flow imaging and aids in the identification of areas triggered by stimuli (Rawnaque et al., 2020). Despite the fact that this technology has a higher spatial resolution than any other currently available technology, its lack of portability, high cost, and low temporal resolution compel researchers to seek out other options. EEG and MEG, on the other hand, are technologies with a better temporal resolution than fMRI but with a lower spatial resolution (Rawnaque et al., 2020). Due to the fact that MEG devices require a shielded environment to detect the brain's very low magnetic fields, they are usually expensive. EEG technology has appealed to the neuromarketing sector as a reasonably inexpensive, well-established, and portable instrument from Krugman's original usage in 1971 (Krugman, 1971). Taken together, EEG analysis as a realistic and efficient tool can aid our understanding of the brain's decision-making processes.

In the past, EEG-based neuromarketing-related studies examined how commercial and its design could affect customers' judgement and buying behavior. Khushaba et al. (2013) used photographs of crackers to study marketing stimulus in three different shapes, tastes, and topping to create a sequence of 57 options. The participants were asked to pick their preferred set and categorize their preferences across all sets. The change in EEG spectral activity that accompanied it was then measured. Yılmaz et al. (2014) investigated shoe images in order to obtain user feedback from EEG signals in terms of dislike or like of the corresponding image. A similar strategy was utilized by Yadava et al. (2017). They used 42 photographs of items in various hues and textures. Researchers then used the Hidden Markov model classifier to extract four features from EEG and classify them. Bastiaansen et al. (2018) used photographs of Bruges tourist attractions to split the participants into two groups. One group viewed 11 min and 42 s of the film “In Bruges” before seeing the images, whereas the other (control group) saw an unrelated 9 min and 23 s movie sample. Subsequently, EEG was used to record the differences in emotional responses within these groups.

One of the most concern in marketing research is how consumers deal with diverse product options depending on their own unique perceptions of advantages and costs. The prefrontal cortex (PFC), which is located in the frontal cortex (FC) of the brain, plays a key role in the underlying processes of human decision-making. Several studies show that the parts of PFC are involved in decision-making processes by weighing the pros and cons of various options and outcomes (Tremblay and Schultz, 1999; Daw et al., 2006). People can either be attracted to or repellent to a stimulus when they interact with it. Currently, researchers are looking into brain activity signals that are linked to an increase in emotional involvement when people interact with marketing stimuli (Langleben et al., 2009; Vecchiato et al., 2010). When people experience a consumer product the blood flow of a particular part increases which is usually captured by fMRI (Telpaz et al., 2015; Rawnaque et al., 2020). Simultaneously, the electrical activity of certain part of the human brain shows distinct pattern like oscillation in frequency which is captured by EEG (Telpaz et al., 2015; Rawnaque et al., 2020). While working with EEG, researchers discovered strong links between people's behavioral systems (both positive and bad) and their consumer actions. The activity of specific anatomical areas connected to emotional processing activity in humans, such as the PFC and FC, could be tracked to gather indirect variables of emotional processing (Davidson and Irwin, 1999). The anatomically and functionally various area of PFC are well-known for its role in emotion formation (Davidson, 2000). According to EEG spectral power analyses, left PFC appears to be a significant component of a brain circuit mediating appetitive approach behavior, while the right PFC appears to be a key component of a neural circuit mediating defensive withdrawal behavior (Davidson, 2000, 2004). Measuring the activity of these regions can thus provide useful information about marketing concepts like perceived value and the brain underpinnings of customer decision-making.

In other studies, researchers used Support Vector Machine (SVM) and K-Nearest Neighbor (KNN) to quantify user preferences for aesthetics displayed as virtual three-dimensional objects, with frequency bands acting as attributes for EEG segmentation into binary classes (Agarwal and Dutta, 2015; Ramadan et al., 2015). Hakim et al. (2018) used SVM to combine EEG measurements with questionnaire responses to identify the more and the less favored parts. The type of classification system used in a BCI system is mostly determined by the application's nature and location. With the recent application of deep learning (DL) in different domain (Mashrur et al., 2019, 2021a; Nazi et al., 2021), Teo et al. (2017) showed the subjects 3D virtual jewelry objects, asked to rate them on a Likert scale, and then categorized EEG signals using deep learning. Again, Aldayel et al. (2020) emphasized the need of spectral valence features to improve prediction accuracy and the merging of classifiers using deep learning to extract features. In another work, Aldayel et al. (2021) measured preference indices to classify like and dislike signals. Authors used data from Yadava et al. (2017) which used only product as stimuli and extracted time-frequency domain features. Using these features, they calculated preference indices which was later used to train multiple machine learning and deep learning (DL) models for the classification of EEG signals. Golnar-Nik et al. (2019) employed LDA and SVM classifiers to assess how effectively EEG signals could distinguish various customer preferences and predict the occurrence of decision-making in another study. Telpaz et al. (2015) published one of the most influential research articles in the field of neuromarketing in 2015. The researchers in this study proposed that EEG may be used to forecast future customer choices based on statistical significance. However, extant literature indicates that, there is hardly any validated and significantly accurate ML framework predicting consumer purchase intention from EEG signals. Moreover, EEG-based research on consumers' affective attitude toward advertising stimuli is almost non-existent in current literature. Therefore, in this study, we propose a ML framework for predicting consumer future choices by linking affective attitude (AA) and purchase intention (PI) from EEG signals.

Firstly, we collect EEG signals from the participants while they view three types of advertisements with three different dominant features: one focused on product features; one centered on endorsement and one centered on promotion or offers. After preprocessing, we extract features from the signals. Afterward, using feature selection techniques, we classify the EEG signals into positive and negative for both AA and PI using SVM-RBF classifier. It should be noted that as previous works only focused on only product stimuli (Telpaz et al., 2015; Yadava et al., 2017; Aldayel et al., 2021), we want to propose a more robust and generalize machine learning framework that can recognize beyond only product. Here, our main focus was to find EEG pattern of the participants for these combined heterogeneous stimuli setting. Therefore, we added endorsement and promotion which we are implementing for very first time. Taken together, we are using product, product + endorsement, and product + promotion to increase the generalizability of the proposed machine learning (ML) framework. Consequently, we combine these stimuli and treat as same while classifying of positive and negative AA and PI. as positive AA (PAA), negative AA (NAA), positive PI (PPI), and negative PI (NPI). The major contributions of this work are listed below.

As previous world only used product image as stimuli,

- To the best of authors' knowledge, this is most likely the first study that propose ML framework for predicting consumers' purchase intention and affective attitude (toward advertising stimuli) from EEG signals.
- We show the EEG signals differences between positive and negative response (AA and PI). In addition, we also report EEG signals differences among the advertising stimuli.
- A thorough experimental evaluation (hyperparameter tuning) is carried out to establish the feasibility of the proposed method.
- We also suggest consumer grade device to implement such Neuromarketing framework in real life setting.

## 2. Materials

This section discusses the research participants, stimuli, and data collection description.

### 2.1. Participants

Twenty healthy young volunteers (age: 24±7.2) participated in this study with no history of neurological or mental disorders. Before the study, According to the Helsinki Declaration and Neuromarketing Science and Business Association Code of Ethics (NMSBA), all participants provided their consent. The study is also approved by the United International University, Institutional Research Ethics Board committee.

### 2.2. Stimuli Description

We use five different items in this research, each with its endorsement and promotion-based (offers) advertisement. A product endorsement is a tool in marketing communication that has a positive effect on customers. In the vast majority of cases, celebrities promote a product in a real-world setting. Nevertheless, in order to avoid biasing the participants, we use neutral endorsement in our research. On the other hand, sales promotion is a technique used by marketers where they give discounts, cashback or any other monetary offers so that the customers are attracted to buy the product. In our case, we offer a buy one get one free or a 50% discount with that product. The stimuli are shown in Figure 2, where each row represents a different product: burgers, sunglasses, cake, hats, and coats. The baseline products are in the first column, endorsements are in the second column, and promotion stimuli are in the third column for each product.

### 2.3. Data Collection

**Figure 1:**
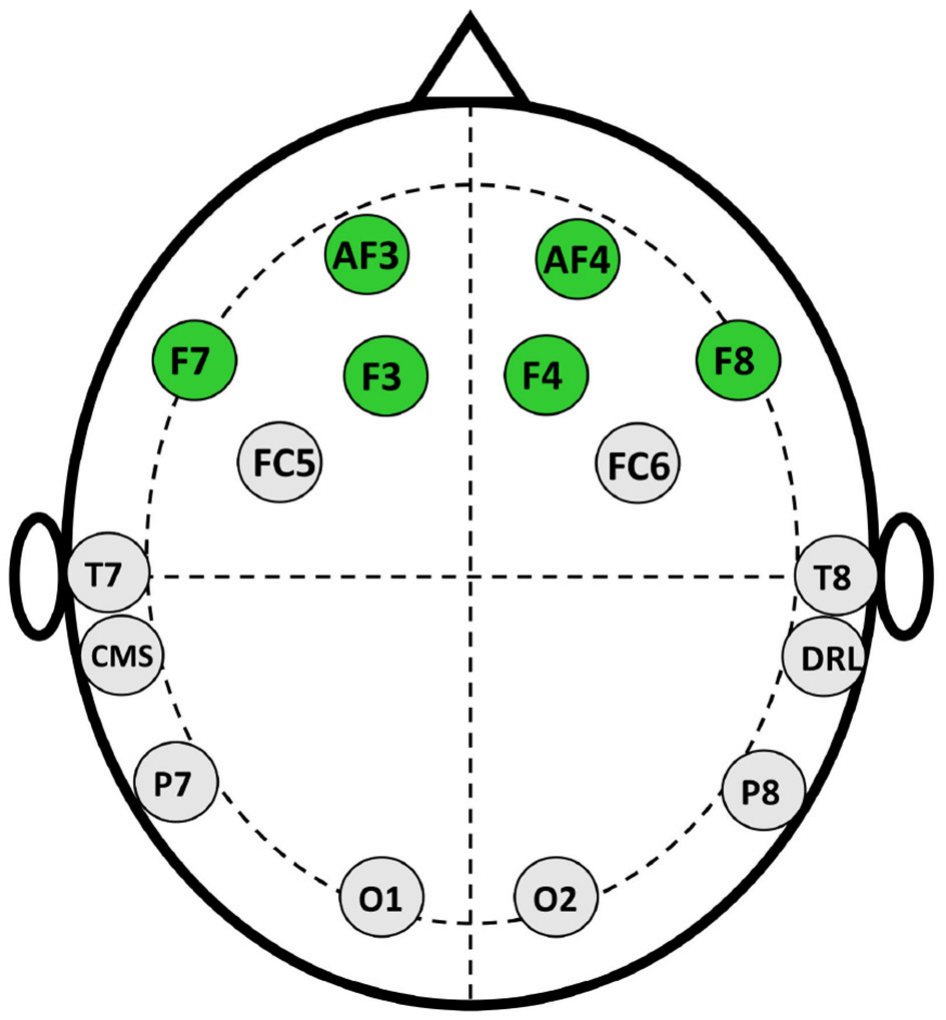
Electrode positioning of EMotiv epoch+ device.

The data collection process is separated into three stages which are inspired by Levy et al. (2011) and Telpaz et al. (2015). In stage 1, the experimenter briefs the participants about the stimuli so that they will be at ease when watching them on screen. We do not show actual stimuli to the participants before the experiments, rather experimenters describe the promotion and endorsement beforehand using a different image that is not used as stimuli. This makes sure the participants watch the stimuli for the first time while collecting EEG signals. In the second stage, participants sit comfortably in front of a monitor that displays the stimuli at a 75–100 cm distance. Then, We set the Emotiv Epoch+ headset (electrode position illustrated in Figure 1) in the participant's head and ensure the electrode conduction is good enough to collect EEG data. After that, we use PsychoPy v3.0 (Peirce, 2007) to show the stimulus to the participants and collect EEG data simultaneously. The sampling rate of EEG signals is 128 Hz. We use six frontal channels for this work because previous studies suggest better performance with FC (Rawnaque et al., 2020; Mashrur et al., 2021b). Illustrated in Figure 3. Throughout the trial, each product was presented for 5 s, followed by an endorsement or promotion. Moreover, before showing each stimulus a black screen is shown with a white plus sign in the middle to keep the focus of the participants on the screen. In stage 3, we gave the participants a questionnaire in which each stimulus is accompanied by the following statements: 1. I would be happy to have x and 2. If given the opportunity, I am willing to buy x. The first question is demonstrating the affective attitude and the second one is purchase intention. Participants respond on a numeric scale of 1–10 (strongly disagree to strongly agree), which was later converted into: negative (1–5) and positive (6–10).

**Figure 2:**
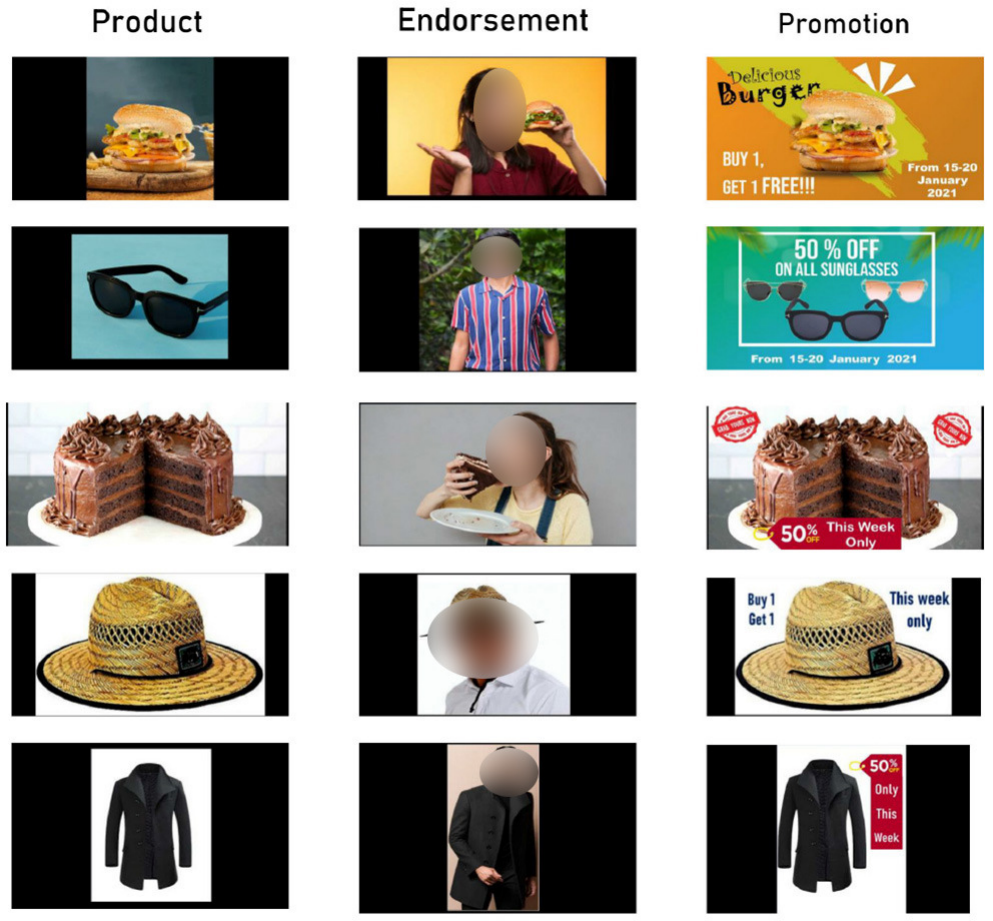
Stimuli used in our experimental setup, with the first column representing the baseline product, the second column depicting endorsement stimuli, and the last column representing promotion stimuli.

**Figure 3:**
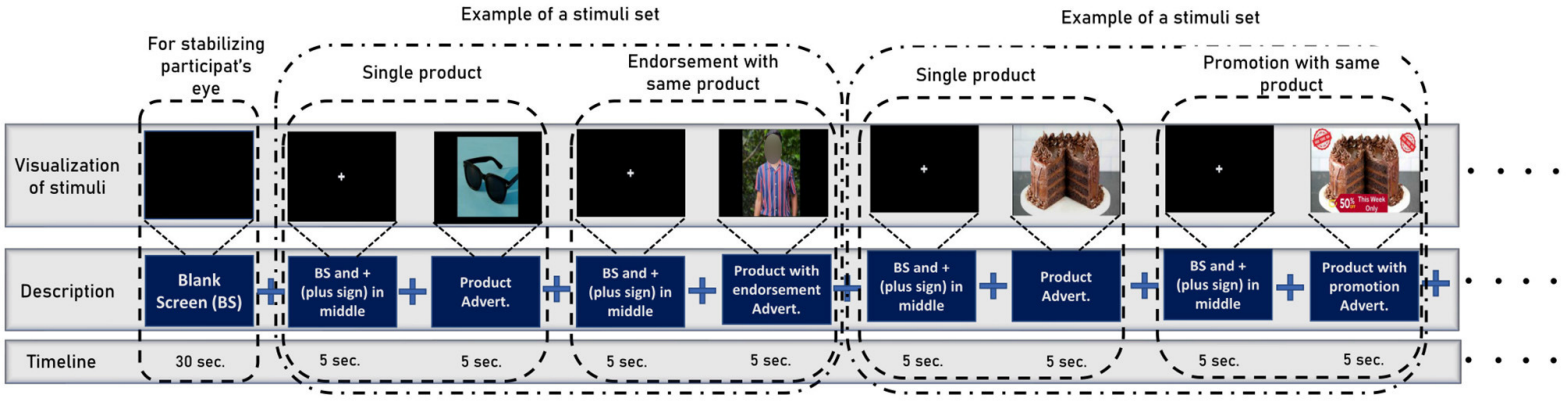
The stimuli sequence while we collect the EEG data from participants. To begin, the participants are shown a blank screen to aid in visual stability. Then, it shows a set of stimuli for a specific product at random intervals (first a product, then its endorsement, or promotion). Note that before showing each stimulus a black screen is shown with a white plus sign in the middle to keep the focus of the participants on the screen.

## 3. Methods

**Figure 4:**
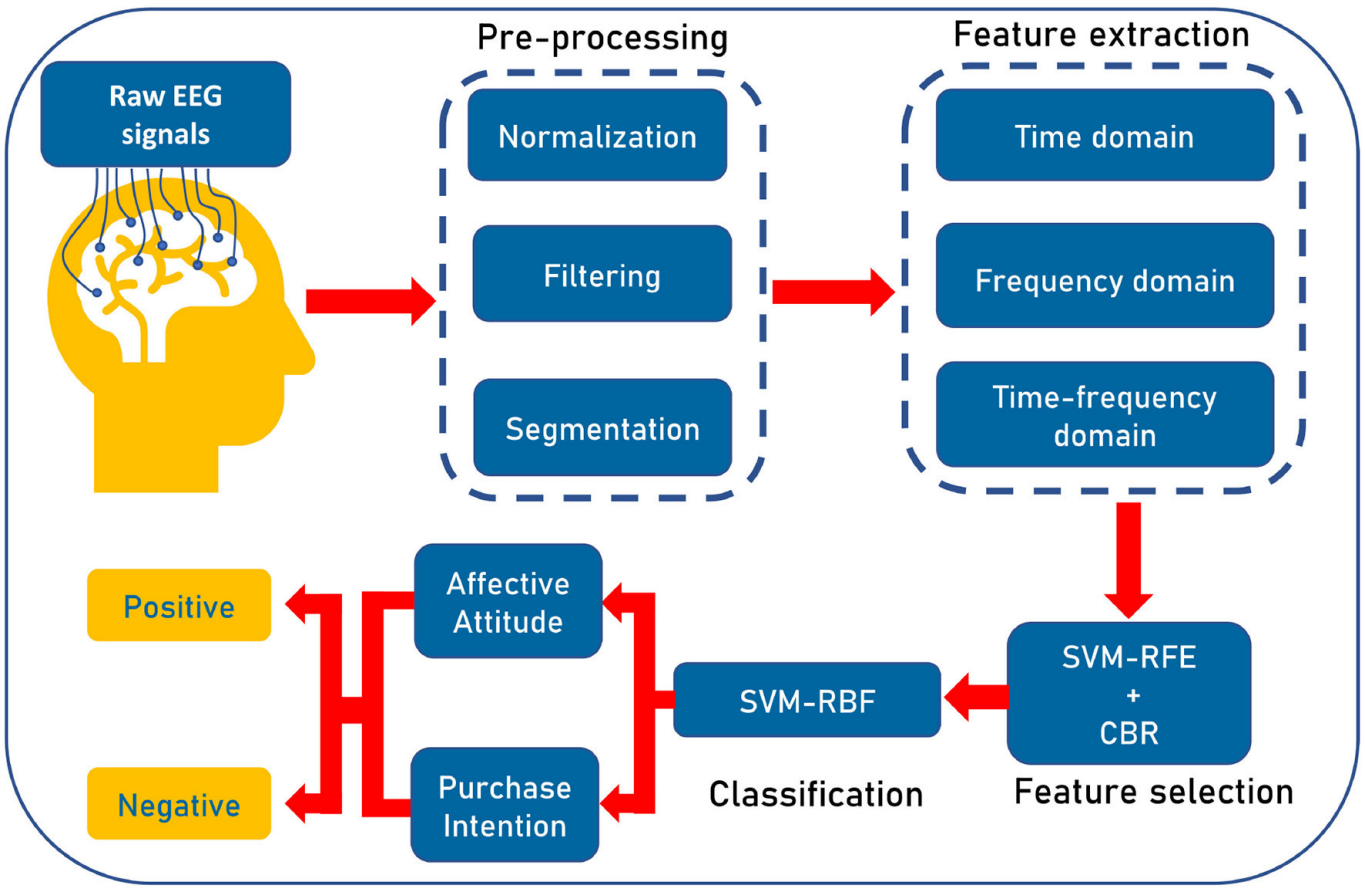
Illustration of the workflow of our proposed pipeline. At first, we preprocess the raw EEG signals to eliminate the noise and prepare the signals. Then three types of features, namely, time, frequency, and time-frequency domain features are extracted. Then, wrapper-based Support Vector Machine-Recursive Feature Elimination (SVM-RFE) along with correlation bias reduction is used for feature selection. Lastly, we use SVM with radial basis function (RBF) kernel for categorizing positive affective attitude and negative affective attitude.

The workflow of our proposed algorithm is illustrated in Figure 4. At first, we preprocess the raw EEG signals to eliminate the noise and then segment the data. Next, we extract features from signals. After that, the best features are selected to classify positive and negative AA and PI using SVM. The following notation is used throughout this paper: The dataset *Y* = [*y*_1_, *y*_2_, ..., *y*_*N*_], where *N* is the number of participants. For any participant *Y*_*N*_, the segmented time series vector for one electrode is *X*(*t*)∈*R*^*T*^, where *T* represents the number of samples in time. Again, the feature matrix is denoted by *F* = [*f*_1_, *f*_2_, ..., *f*_*t*_], where *f*_*t*_ is the vector (all samples) for a feature and *t* is the total number of features.

### 3.1. Pre-processing

Both customized scripts in MATLAB 2020a (MathWorks, Natick, MA) and EEGLAB (Delorme and Makeig, 2004) are used to preprocess EEG signals. *Y*_*N*_ is first normalized by subtracting the mean from all sample points and dividing each point by the standard deviation. The power line noise is then removed using a notch filter (50 Hz). The signals are then filtered using a third-order Butterworth bandpass filter with a frequency range of 0.5–48 Hz to remove high and low-frequency noise. Furthermore, Independent component analysis (ICA) (Hyvärinen and Oja, 2000) is used to distinguish the source of the signals and eliminate noise such as eye blink, electrocardiogram, muscle movement, and line noise. Finally, *Y*_*N*_ are segmented and averaged (participants wise), resulting in our structured *X*(*t*) time series vector.

### 3.2. Feature Extraction

This subsection describes the extracted features for this study, categorized by: time domain, frequency domain, and time-frequency domain features similar as Mamun et al. (2015). Table 1 shows the feature list used in this work. According to the literature (Section 1) PFC and FC are mostly responsible for AA and PI (Davidson and Irwin, 1999; Davidson, 2000, 2004; Ramsøy et al., 2018). Therefore, we extract feature from FC in this work. When people watch a stimulus neurons within a certain brain become active and pass a small electric current which can be detected placing a sensor on the human scalp (Luck, 2014). Again, according to Davidson (2000) active area of the FC varies with corresponding neuromarketing stimuli. According to the extent literature, for the first time, we use ML based neuromarketing work that uses endorsement and promotion as stimuli. Therefore, we use a diverse feature set to capture any changes in the electrical signal of the brain. So, this work represents those features that captures distinct firing pattern from EEG signals. Previously, the power and some statistical features were widely used in Neuromarketing works (Golnar-Nik et al., 2019; Yadava et al., 2017). Along with these, we increased the feature set to capture more subtle changes in the EEG for the mixed stimuli setting. From *f*_1_ to *f*_11_, we use statistical and power features that were before for EEG signals pattern recognition (Inuso et al., 2007; Islam et al., 2013; Ahammad et al., 2014; Jenke et al., 2014; Golnar-Nik et al., 2019). Moreover, this study finds dispersion to be prominent feature for Neuromarketing, therefore we use features from *f*_1_2 to *f*_20_. In addition, literature shows that, frequency band oscillation and spectral changes are significant while measuring EEG for decision making, attention, and consumer choice, consequently, we also used diverse spectral feature for this study (Foxe and Snyder, 2011; Nácher et al., 2013; Telpaz et al., 2015; Mashrur et al., 2021b).

**Table 1:** List of base features used in this work.

**Index**	**Feature name**	**Description**
**f*_1_*	Average power (Golnar-Nik et al., 2019)	Mean power of EEG calculated by power spectra density (PSD) using Welch's method
**f*_2_*	Relative power (Golnar-Nik et al., 2019)	Band power over total power of the EEG signals
**f*_3_*	Hjorth mobility (Jenke et al., 2014)	Hjorth feature
**f*_4_*	Hjorth complexity (Jenke et al., 2014)	Hjorth feature
**f*_5_*	Skewness (Islam et al., 2013)	Degree of symmetry of EEG signals
**f*_6_*	Arithmetic mean (Jenke et al., 2014)	Mean value of EEG signals
**f*_7_*	Median value (Islam et al., 2013)	Median value of EEG signals
**f*_8_*	Minimum value (Islam et al., 2013)	Lowest Value of EEG signals
**f*_9_*	Mean absolute value (Phinyomark et al., 2012)	Mean absolute value of EEG signals
**f*_10_*	Interquartile range (Ahammad et al., 2014)	Difference between 75*th* percentiles and 25*th* percentiles
**f*_11_*	Renyi entropy (Inuso et al., 2007)	Non-linear entropy of EEG signals
**f*_12_*	Absolute threshold crossing (Tkach et al., 2010)	Number of times EEG signals cross threshold value:*T*_1_ = 0.5
**f*_13_*	Threshold crossing (Toledo-Pérez et al., 2020)	Number of times EEG signals cross threshold value: $T_{2}=4\times \frac{1}{10}\overset{10}{\underset{i=1}{\sum }}X\left(i\right)$
**f*_14_*	Zero crossing (Jenke et al., 2014)	Number of times EEG signals changes sign
**f*_15_*	Slope sign change (Sharmila and Geethanjali, 2018)	Number of times EEG signals change slope sign
**f*_16_*	Square integral (Phinyomark et al., 2012)	Summation of square EEG signals
**f*_17_*	Log detector (Phinyomark et al., 2012)	Non-linear natural exponential measurement
**f*_18_*	Cardinality (Waris and Kamavuako, 2018)	Number of distinct value
**f*_19_*	Autoregressive model (Zhang et al., 2017)	Linear regression of the present EEG signals observation against one or more preceding series data
**f*_20_*	Detrend fluctuation analysis (Oon et al., 2018)	Non-linear measure of auto-correlation properties
**f*_21_*	Spectral centroid (Peeters, 2004)	Barycenter of the spectrum
**f*_22_*	Spectral spread (Peeters, 2004)	Spread of the spectrum around its mean value
**f*_23_*	Spectral kurtosis (Peeters, 2004)	Flatness distribution of spectrum around its mean value
**f*_24_*	Spectral entropy (Misra et al., 2004)	Peakiness distribution of the spectrum
**f*_25_*	Spectral flatness (Johnston, 1988)	Noise like nature of the spectrum
**f*_26_*	Spectral crest (Peeters, 2004)	Sinusoidality of the spectrum
**f*_27_*	Spectral slope (Peeters, 2004)	Linear decreasing of the spectral amplitude
**f*_28_*	Spectral decrease (Peeters, 2004)	Decreasing of the spectral amplitude
**f*_29_*	Spectral rolloff point (Scheirer and Slaney, 1997)	95th percentile of the spectral power distribution

#### 3.2.1. Time Domain Features (TDFs)

TDFs are calculated from *X*(*t*) decomposed in time domain. Here, as mentioned in Table 1, feature index *f*_1_ to *f*_20_ are used as TDFs.

#### 3.2.2. Frequency Domain Features (FDFs)

FDFs are extracted to find changes in the frequency domain of *X*(*t*). In this work, we estimate spectral features (SFs) described in Table 1 as feature index *f*_21_ to *f*_29_. In this study, the average value of the SFs obtained with MATLAB 2020a. Despite their ubiquitous usage in speech and audio signal classification, SFs have recently been used for EEG signal categorization (Hassan and Subasi, 2016; Rashid et al., 2018). SFs record the amplitude spectrum of EEG data, which gives discriminating information between classes.

#### 3.2.3. Time-Frequency Domain Features (TFDFs)

EEG signals are complicated in that they have qualities in both the temporal and frequency domains. EEG signals are split into six bands in this work utilizing wavelet packet transformation (WPT), which may recover frequency information without leaving the temporal domain. In the literature, WPT has been routinely utilized to distinguish frequency bands from EEG data (Wali et al., 2013; Vidyaratne and Iftekharuddin, 2017; Phanikrishna and Chinara, 2021).

##### 3.2.3.1. WPT

The signal is decomposed by WPT into both detailed and approximate coefficients. The extracted coefficients up to a defined level could be considered as features because their values are dependent on the time and frequency domain characteristics of the EEG signals. WPT creates a subspace tree of a signal with distinct frequency characteristics by recursively applying high-pass and low-pass filters (Percival and Walden, 2000). Let, $\prod_{a,b}\left(k\right),n=0,..,2^{a}-1$, denote the WPT coefficients at level *a*. The wavelet packet coefficients are then computed using the two wavelet packet orthogonal bases equations:


(1) $$\begin{array}{l} \begin{array}{c} \prod_{a,2b}\left(i\right)=\sum_{l=0}^{L-1}H\left(s\right)\prod_{a-1,b}\left(2k+1-\mathrm{lmod}N_{b-1}\right) \end{array} \end{array}$$

(2) $$\begin{array}{l} \begin{array}{c} \prod_{a,2b+1}\left(i\right)=\sum_{l=0}^{L-1}G\left(s\right)\prod_{a-1,b}\left(2k+1-\mathrm{lmod}N_{b-1}\right) \end{array} \end{array}$$

Where *H*(*s*) and *G*(*s*) are the impulse responses which are highpass and lowpass filters of the wavelet packets respectively and *i* = 1…*N* and *N*_*b*_ = *N*/2_*b*_ (Percival and Walden, 2000). Here, we use Meyer wavelet for computing the sub-bands as it showed better performance in previous work (Mamun, 2011) using EEG signals.

To extract TFDFs, *X*(*t*) is decomposed in five levels extracting six bands, namely, δ = 0−4Hz, θ = 4−8Hz, α = 8−12Hz, β_1_ = 12−20Hz, β_2_ = 20−32Hz, γ = 32−64Hz. Then all the features, as mentioned in Table 1, were extracted from each band, with a total of 246 features. Again, as power features performed well in the literature (Golnar-Nik et al., 2019), ratio of average and relative power also calculated as separate features. The ratios are: $\frac{\theta }{\delta },\frac{\alpha }{\delta },\frac{\beta_{1}}{\delta },\frac{\beta_{2}}{\delta },\frac{\gamma }{\delta },\frac{\alpha }{\theta },\frac{\beta_{1}}{\theta },\frac{\beta_{2}}{\theta },\frac{\gamma }{\theta },\frac{\beta }{\alpha },$ $\frac{\beta_{2}}{\alpha },\frac{\gamma }{\alpha },\frac{\beta_{2}}{\beta_{1}},\frac{\gamma }{\beta_{1}},\frac{\gamma }{\beta_{2}}$.

### 3.3. Feature Selection and Classification

To select the best feature set, we use wrapper-based Support Vector Machine-Recursive Feature Elimination (SVM-RFE) with correlation bias reduction (CBR) (Yan and Zhang, 2015). Initially, Guyon et al. (2002) propose SVM-RFE which evaluates features using criteria derived from SVM model coefficients, then recursively eliminates features with small criteria. This method can be used in both linear and nonlinear situations (Guyon et al., 2002; Rakotomamonjy, 2003). When the optimal decision function is nonlinear, the nonlinear SVM-RFE approach is preferred since it incorporates a novel kernel method. We also employed a non-linear variant with a radial basis function (RBF) as the kernel in our research. For its backward elimination method, SVM-RFE may represent feature dependencies. SVM-RFE does not use cross-validation (cv) accuracy on the training data as a selection criterion, which means it is less prone to overfitting, can fully utilize the training data, and has a substantially shorter execution time, especially when there are a high number of candidate features. As a result, it's been applied to a range of problems, such as gene selection (Guyon et al., 2002; Rakotomamonjy, 2003; Duan et al., 2005; Mundra and Rajapakse, 2009). However, when some of the features are highly associated, the assessing criteria of these features would be altered by underestimating their value. To address this, inspired by Toloşi and Lengauer (2011) and Yan and Zhang (2015) suggested a robust technique, SVM-RFE + CBR, for estimating gas sensor characteristics.

In this work, SVM is used for classification. The SVM identifies the appropriate boundary in the feature space where the model is trained using the structural risk reduction criterion by combining a maximal margin approach with a kernel method (Gunn et al., 1998; Hart et al., 2000). SVM utilizes a kernel function to translate the inputs into a high-dimensional feature space, whereas learning derives the decision boundaries directly from the training data set. Then, it constructs an optimal separation hyperplane in the feature space. The choice of an appropriate kernel function is crucial for optimizing SVM classifier performance (Gunn et al., 1998). We use RBF kernel for it's better performance earlier research based on EEG signals (Li et al., 2014; Zainuddin et al., 2018; Anuragi and Sisodia, 2019). To increase classification performance, an SVM classifier's hyper-parameters, notably the regularization parameter *C* and the *gamma*, are tuned during training as demonstrated most efficient and accurate in Hsu et al. (2003). We utilize the LIBSVM (Chang and Lin, 2011) function to classify our work. In addition, we use other classifiers too namely, Naive Bayes, Decision tree, K-Nearest Neighbor (Bonaccorso, 2017).

### 3.4. Metrics for Assessing Performance

In order to access the performance of our proposed pipeline, several metrics, namely, accuracy (Acc.), sensitivity (Sens.), and specificity (Spec.).


(3) $$\begin{array}{l} \begin{array}{c} \text{Sens}=\frac{TP}{TP+FN} \end{array} \end{array}$$

(4) $$\begin{array}{l} \begin{array}{c} \text{Spec}=\frac{TN}{TN+FP} \end{array} \end{array}$$

(5) $$\begin{array}{l} \begin{array}{c} \text{ACC}=\frac{TP+TN}{TP+TN+FN+FP} \end{array} \end{array}$$

where *FP, FN, TP*, and *TN* are the number of false positives, false negatives, true positives, and true negatives, respectively. We report the mean value of the metrices. The Shapiro-Wilk test is used to determine the normality of the data. Moreover, we use Wilcoxon Signed-Ranked Test for non parametric hypothesis testing. Again, Friedman test is used for matched, non parametric data to find differences among advertisements.

## 4. Results

This section represents the results obtained by the proposed model. We report the performance of channel pairs for both PI and AA. In addition, We present the report the dispersion differences between PAA and NAA. Furthermore, we report the most contributing feature domain and EEG bands while classifying the signals.

In this work, we use leave-one-subject-out(LOSO) evaluation techniques where the features are separated in 20 as the total number of subjects is 20. Every subject is used for the test set only once, while the rest is used as the training set.

**Figure 5:**
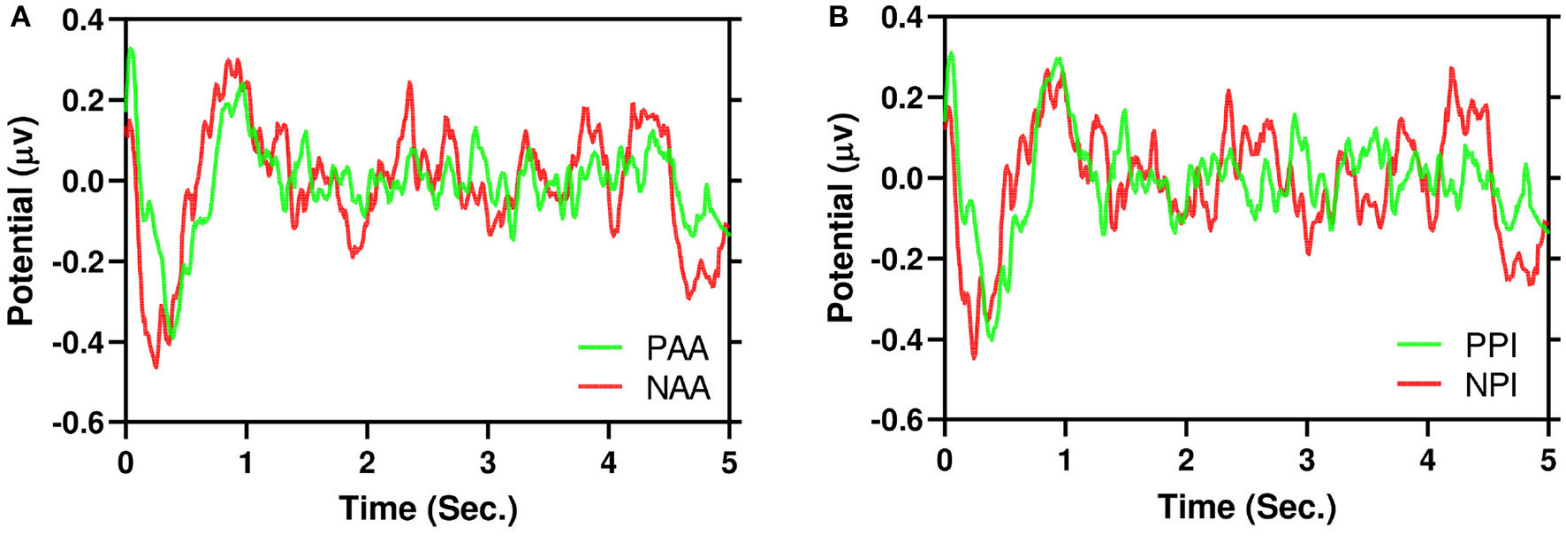
Example of grand average 5 s EEG signals in time domain for *AF*3 channel. It is evident that negative (red) signals have higher dispersion than positive (green). **(A)** AA. **(B)** PI.

Figure 5A illustrates the grand average of positive AA and negative AA in the time domain for *AF*3 channel. Similarly, Figure 5B also illustrates the same for PI. It is evident that negative and positive signals have N200 to N400 components respectively. Moreover, to test dispersion of negative and positive signals, a Wilcoxon Signed-Ranked Test (WSRT) indicates that the standard deviation of NAA is significantly higher than the standard deviation of PAA *Z* = 133, *p* = 0.0006. However, WSRT does not find dispersion between NPI and PPI.

**Figure 6:**
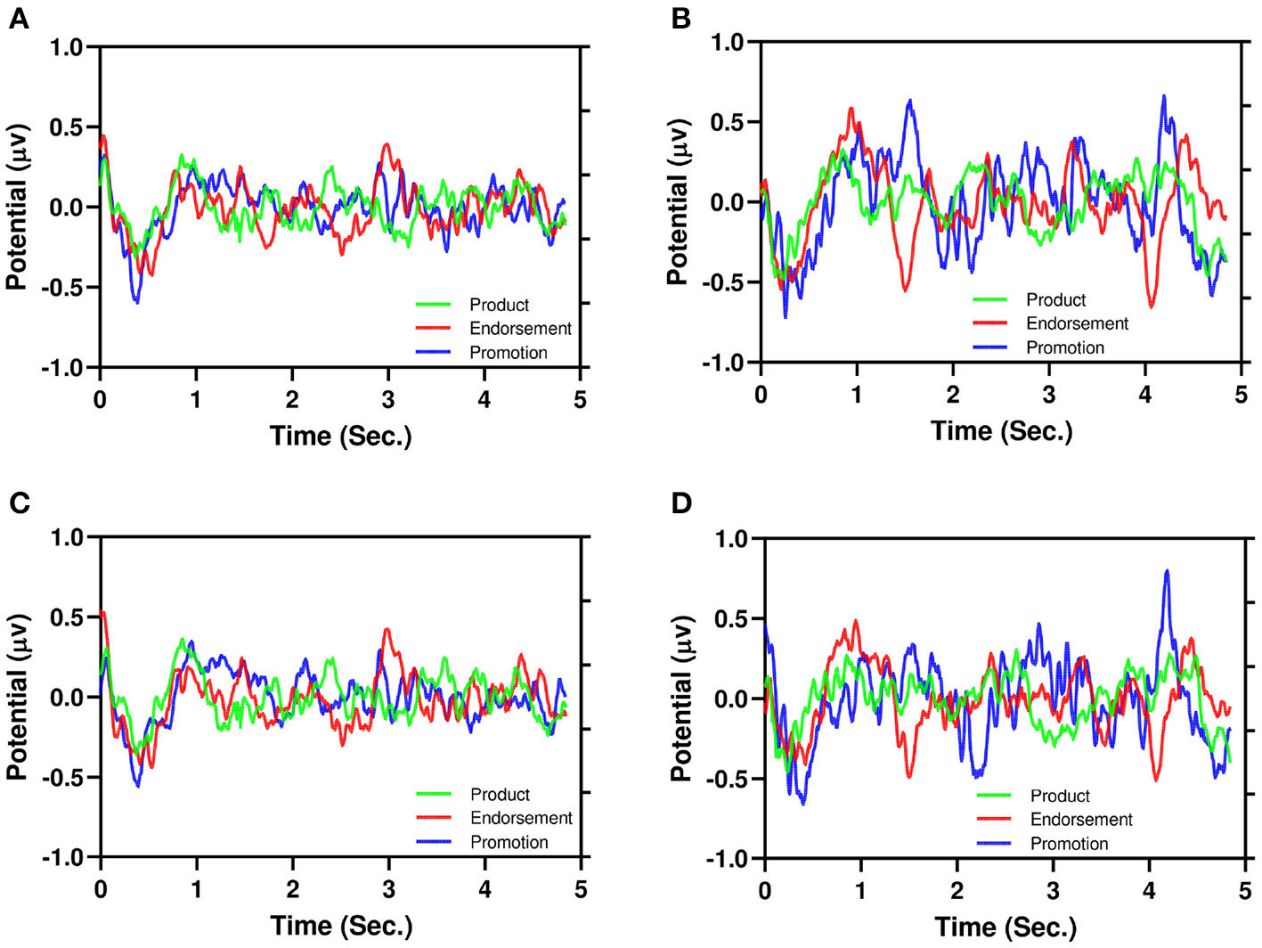
Illustration of the average of EEG signals of product, endorsement, and promotion. **(A)** PAA, **(B)** NAA, **(C)** PPI, **(D)** NPI.

Figure 6 illustrates the average of EEG signals of product, endorsement, and promotion: Figure 6A (PAA), Figure 6B (NAA), Figure 6C (PPI), and Figure 6D (NPI). It is also evident from the PAA and PPI (Figures 6A,C) have less dispersion than NAA and NPI (Figures 6B,D) similar to Figure 5. Moreover, for NAA and NPI (Figures 6B,D), three advertising stimuli showed different EEG signatures (around 1.5–4.0 s). For endorsement, there is a negative peak and for promotion, there is a positive peak. However, the product shows neutrality in the signals.

Again, in Table 2, we report the performance of our proposed model for four combinations. We take the first three symmetrical channels as pairs from EEG montage, namely AF3+AF4, F3+F4, and F7+F8. The fourth combination is all FC channels (combination of three pairs). The combination of all channels gives the best results of accuracy 84.00 and 87.00% for PI and AA, respectively. In addition, among three pairs F3+F4 performs the best with an accuracy of 81.50 and 85.50% for PI and AA, respectively. For all the combinations, sensitivity is higher than specificity meaning that the true positive is high in our proposed framework. We also use other classification methods for all FC channels. Naive Bayes, Decision Tree, K-Nearest Neighbors yields 67.14, 70.24, 72.14% for PI and 70.78, 72.62, 73.33% for AA, respectively.

**Table 2:** Performance of our proposed framework.

**Channel**	**PI**			**AA**		
	**Acc. (%)**	**Spec. (%)**	**Sens. (%)**	**Acc. (%)**	**Spec. (%)**	**Sens. (%)**
AF3+AF4	80.00	71.52	85.53	84.75	71.31	91.14
F3+F4	81.50	77.21	84.30	85.50	72.09	91.53
F7+F8	79.00	72.78	83.05	82.50	65.12	90.77
AF3+AF4+ F3+F4+F7+F8	84.00	75.32	89.66	87.00	74.41	92.98

**Figure 7:**
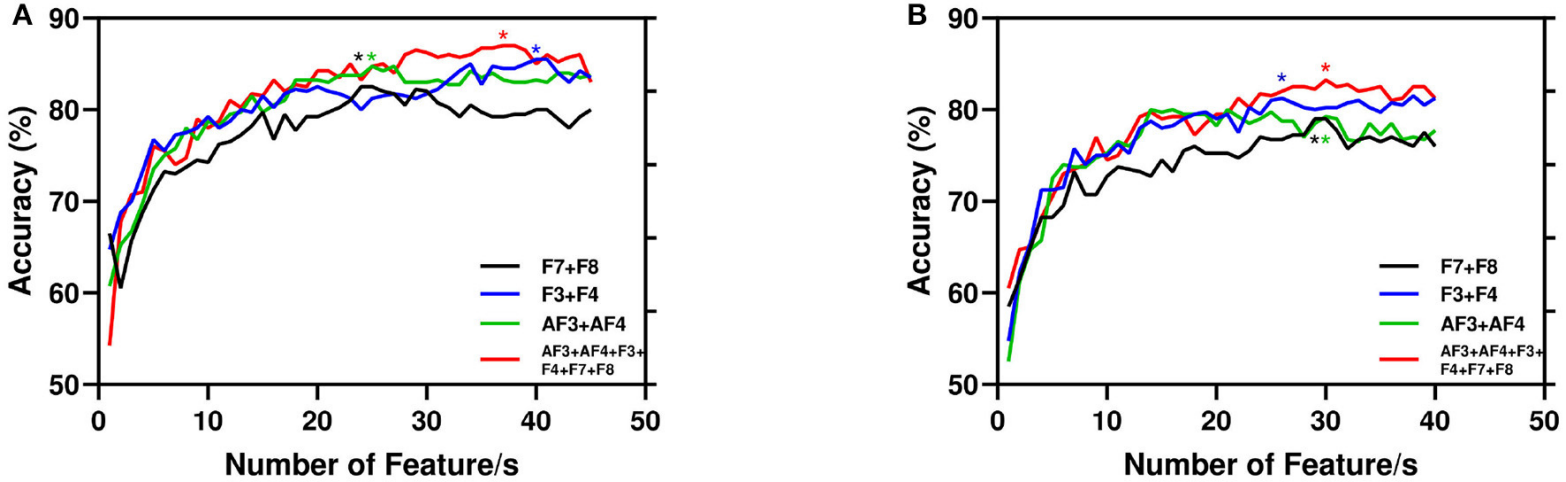
Performance of the proposed model with number of features. Here, for channel combination best results are started (*) marked with respective color vertical to number of features. **(A)** AA. **(B)** PI.

Again, Figure 7 depicts the accuracy with respect to the number of features. Here, we present the same combination of channels reported in Table 2. It is evident that the performance of the model improves with the number of features. However, for AA the experimented total number of features is 45 as the increasing features more than that leads to poor performance. Again, for PI we report the highest 40 features as the performance stabilized around 35 features.

We also evaluate the difference of AA and PI among three advertisements, namely, product, endorsement, and promotion for both reported (reported outcome from the participant) and predicted (the outcome we get from our proposed framework). Friedman test of differences among repeated measures (reported outcome of three advertisements for AA) is conducted which yield a Chi-square value of 20.86 which was significant (*P* < 0.0001). The exact same test for predicted outcome result a Chi-square value of 12.50 which was significant (*P* = 0.0019). Again, for PI, Friedman test for reported outcome give a result of Chi-square value of 15.92 which was significant (*P* = 0.0003). Moreover, for predicted outcome the Chi-square value of 6.75 which was significant (*P* = 0.0343).

**Figure 8:**
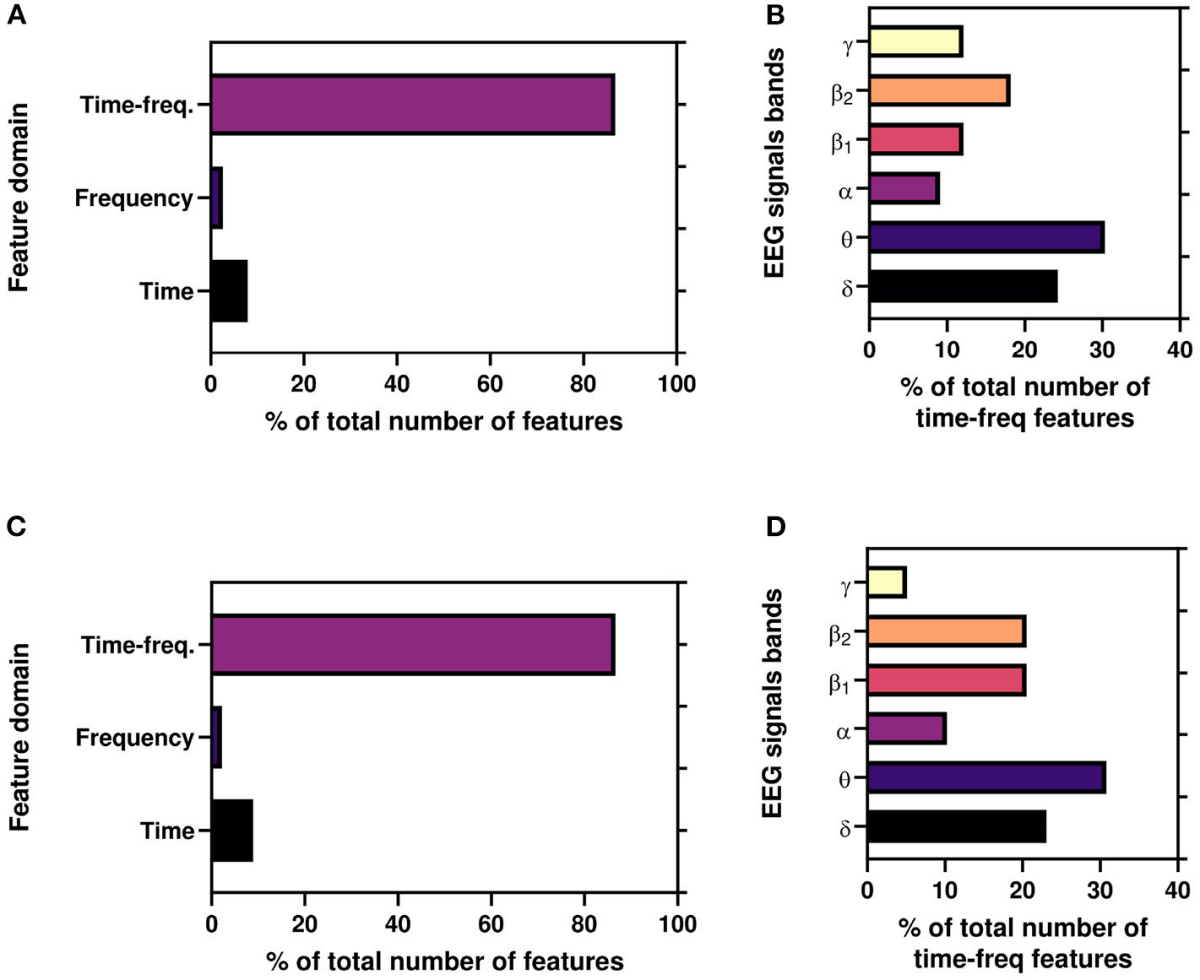
**(A,C)** Illustrate the percentage of features(domain wise) for working results reported in Table 2. **(B,D)** Illustrate the percentage of time-frequency domain features (band wise) for best results reported in Table 2.

Again, Figure 8 depicts the percentage of features(domain wise) for best results reported in Table 2 for both AA and PI. Here, time-frequency domain features dominate the most significant features in the classification task. Again, we also illustrate the percentage of time-frequency domain features for six bands. Here, it can be seen that theta (θ) band mostly dominates followed by delta (δ), beta2 (β_1_), and beta1(β_2_) bands.

## 5. Discussion

In this study, we demonstrate a framework to classify consumer choice from EEG signals. This is the initial work that predicts the purchase intention (PI) using the ML framework. In addition, this is most likely the first research adopting advertising stimuli as affective attitude (AA) prediction.

For the first time, we proposed that NAA has higher EEG signals dispersion than PAA which is illustrated in Figure 5. In addition, Figure 6 depicts that positive AA and PI (Figures 6A,C) have less dispersion than negative AA and PI (Figures 6B,D). Moreover, from Figure 5, it is evident that EEG signals show a negative peak after watching advertising stimuli. It should be mentioned that both NAA and NPI have the peak in N200 where the PAA and PPI show the peak in N400 which is aligned with previous works (Telpaz et al., 2015). Taken together, this indicates that subjects tend to decide NAA and NPI faster than PAA and PPI. Then, after taking the decisions subjects still think or revisit their decision about the negative attitude toward particular products which makes the EEG signals more dispersed than a positive attitude.

Moreover, to the best of authors knowledge, for the first time, we use time, frequency, and time-frequency domain features for the ML framework for the Neuromarketing application. It is evident from Figures 8A,B that subjects' EEG signals are most susceptible to the time-frequency domain which indicates that while choosing a product EEG signals shift among bands. Again, Figures 8B,D refer that θ band is the most significant TFDFs for both AA and PI as supported by previous studies (Telpaz et al., 2015; Rawnaque et al., 2020; Mashrur et al., 2021b). Interestingly, δ band is the second most used feature and for the first which is an unique finding in context of Neuromarketing research. According to a previous study, δ band is responsible for decision-making (Nácher et al., 2013) which may explain the significance of δ band in our study. Further study is needed to explore this band's importance.

Our methods improve the level of generalization by increasing the number of subjects along with rigorous hyperparameter tuning with the SVM RBF kernel. We chose wrapper-based SVM-RFE with CBR which uses an SVM classifier while selecting the best set of features. This method removes the highly correlated features first and then ranks the features based on the SVM-RBF kernel. We tune the α and *C* parameters in the kernel to find the best working models using the LOSO evaluation technique. As the feature selection is wrapper-based, this uses a classifier while selecting the feature set. We also use other classifiers such as Naive Bayes, Decision Tree, K-Nearest Neighbors which yield the accuracy of both AA and PI around 67–73% which is compared low compared to current results. Taken together, our model ensures a robust classification that works best with the SVM-RBF kernel classifier.

Our proposed framework also simulates the real-life results which are proved by significant results in the ANOVA test (Friedman test). At first, we test the reported results for three advertisements for both AA and PI which give significantly different results. Afterward, we perform the same with the predicted results and it is also yielding significant results.

Again, according to Figures 6B,D, NAA and NPI show that promotion stimuli trigger a positive peak around 1.5 and 4 s while endorsement stimuli trigger a negative peak. However, for product stimuli the EEG signals do not show any peak. This can be explained by promotion having a 50% off which creates the positive peak and endorsers bring the negative peak. Note that, PAA and PPI do not show any kind of peak for advertising differentiation. For additional investigation, a future study with simultaneous eye tracking is required.

Lastly, this work paves the way for implementing such a neuromarketing framework using consumer-grade EEG devices (CEEGDs) in a real-life setting. The most commonly used CEEGDs (provided channel/s) are Emotiv Insight (*AF*_3_, *AF*_4_, *P*_*z*_, *T*_7_, *T*_8_), Neurosky Mindwave 2 (*Fp*_1_), Muse 2 (*AF*_7_, *AF*_8_), FocusCalm (*F*_*p*_*Z*). According to our result, Emotiv Insight can be a good choice for practical application as this gives comparatively better performance. Though due to our device limitation (Emotive epoch + does not has *F*_*p*_*Z* channel), we are not able to measure the performance of the channel *F*_*p*_*Z*, Focus Calm can be an interesting choice for future researchers to explore it's potential. Based on the search for available devices in the market, no CEEGDs offer *F*3, *F*4 channels. An integration of these channels in CEEGDs will improve their performances as supported by the findings of our research. Nevertheless, the performance will be largely dependent on the sensors of each device and the quality of the raw EEG signals.

## 6. Conclusion

This research presents a comprehensive machine learning framework to classify EEG signals based on consumers' future choices: affective attitude and purchase intention. We also propose that a negative attitude has higher dispersion and faster response. In addition, TFDFs have mostly used features in our proposed framework. Moreover, the proposed model is also able to replicate real-life reported results. In the future, researchers can work on different types of endorsements such as neutral endorsement and celebrity endorsement. Participants in this study are limited to young adult subjects considering them as target consumers of the marketing stimuli. In future, a more diverse subject group may be included alongside different intervals of purchase like daily required products, weekly or monthly, and product-groups like fresh, stationery, home or office appliances, etc. The future researchers may also add more features and fine-tune the classifier to improve the single-channel performance. Lastly, it is evident that neuromarketing is efficient in forecasting consumer preferences and behaviors.

## Data Availability Statement

The raw data supporting the conclusions of this article will be made available by the authors, without undue reservation.

## Ethics Statement

The studies involving human participants were reviewed and approved by Institutional Research Ethics Board, United International University. The patients/participants provided their written informed consent to participate in this study.

## Author Contributions

KM, FM, KR, MM, FS, RV, and SA contributed to conception and design of the study. KR, MM, and KM revised the draft of the manuscript. FM performed the formal analysis and illustration and wrote the first draft of the manuscript. KM supervised and administered the project. All authors contributed to manuscript revision, read, and approved the submitted version.

## Funding

This study was funded by Market-Brain: A Neuromarketing System for Advanced Market Research Project Under ICT innovation Fund, ICT Division, MoPTIT, GOB, Project Code No. 1280101-120008431-3631108 and Institute for Advanced Research, United International University, Bangladesh (Code: IAR/01/19/SE/10).

## Conflict of Interest

The authors declare that the research was conducted in the absence of any commercial or financial relationships that could be construed as a potential conflict of interest.

## Publisher's Note

All claims expressed in this article are solely those of the authors and do not necessarily represent those of their affiliated organizations, or those of the publisher, the editors and the reviewers. Any product that may be evaluated in this article, or claim that may be made by its manufacturer, is not guaranteed or endorsed by the publisher.
